# Mechanistic in vitro studies confirm that inhibition of the renal apical efflux transporter multidrug and toxin extrusion (MATE) 1, and not altered absorption, underlies the increased metformin exposure observed in clinical interactions with cimetidine, trimethoprim or pyrimethamine

**DOI:** 10.1002/prp2.357

**Published:** 2017-09-21

**Authors:** Robert Elsby, Stephen Chidlaw, Samuel Outteridge, Sarah Pickering, Amy Radcliffe, Rebecca Sullivan, Hayley Jones, Philip Butler

**Affiliations:** ^1^ Drug Transporter Sciences Cyprotex Discovery Ltd (an Evotec company) No 24 Mereside, Alderley Park Macclesfield Cheshire United Kingdom; ^2^ Present address: Decision Resources Group The Lexicon Mount Street Manchester United Kingdom

**Keywords:** Drug‐drug interaction, MATE1, metformin, OCT2, pharmacokinetics

## Abstract

Metformin is a common co‐medication for many diseases and the victim of clinical drug‐drug interactions (DDIs) perpetrated by cimetidine, trimethoprim and pyrimethamine, resulting in decreased active renal clearance due to inhibition of organic cation transport proteins and increased plasma exposure of metformin. To understand whether area under the plasma concentration–time curve (AUC) increases relate to absorption, in vitro inhibitory potencies of these drugs against metformin transport by human organic cation transporter (OCT) 1, and the apical to basolateral absorptive permeability of metformin across Caco‐2 cells in the presence of therapeutic intestinal concentrations of cimetidine, trimethoprim or pyrimethamine, were determined. Whilst all inhibited OCT1, none enhanced metformin's absorptive permeability (~0.5 × 10^−6^ cm/sec) suggesting that DDI AUC changes are not related to absorption. Subsequently, to understand whether inhibition of renal transporters are responsible for AUC increases, in vitro inhibitory potencies against metformin transport by human OCT2, multidrug and toxin extrusion (MATE) 1 and MATE2‐K were determined. Ensuing IC_50_ values were incorporated into mechanistic static equations, alongside unbound maximal plasma concentration and transporter fraction excreted values, in order to calculate theoretical increases in metformin AUC due to inhibition by cimetidine, trimethoprim or pyrimethamine. Calculated theoretical fold‐increases in metformin exposure confirmed solitary inhibition of renal MATE1 to be the likely mechanism underlying the observed exposure changes in clinical DDIs. Interestingly, clinically observed increases in metformin AUC were predicted more closely when the renal transporter fraction excreted value derived from oral metformin administration, rather than intravenous, was utilized in theoretical calculations, likely reflecting the “flip‐flop” pharmacokinetic profile of the drug.

**Abbreviation**
AUC: area under the plasma concentration–time curve
BCA: bicinchoninic acid
*C*_max_: maximum plasma concentration
DDI: drug‐drug interaction
ƒ_e_: fraction excreted value
IC_50_: inhibitory concentration that produces 50% inhibition
*K*_i_: absolute inhibition constant
MATE: multidrug and toxin extrusion
OCT: organic cation transporter

## Introduction

Metformin is a biguanide drug used for the treatment of type 2 diabetes and is becoming a common comedication in patients due to the prevalence of diabetes as a comorbidity in many disease areas. As an organic cation at physiological pH, metformin has minimal passive membrane permeability and therefore requires active transport to cross membranes in order to gain entry into cells. Following absorption, metformin is principally eliminated as unchanged drug via active renal tubular secretion into urine (Graham et al., 2011). From three intravenous human mass balance studies, an overall mean of 88 % of total metformin plasma clearance is attributed to renal elimination (Sirtori et al. 1978; Pentikäinen et al. 1979; Tucker et al. 1981). Additionally, based on metformin renal clearance values reported from eight clinical studies (3 mass balance above and 5 DDI), on average 75 % of metformin renal clearance is due to active transporter processes (deriving a fraction excreted value, ƒ_e_, of 0.66) and 25% due to passive filtration (120 mL/min; GFR × *f*_u _= 1) (Somogyi et al. 1987; Wang et al. 2008; Kusuhara et al. 2011; Grün et al. 2013; Müller et al. 2015). The transporters responsible for the active renal elimination of metformin are organic cation transporter (OCT) 2, located on the basolateral membrane of renal proximal tubule epithelial cells, and multidrug and toxin extrusion (MATE) 1/2‐K, located on the apical membrane of tubule epithelial cells, which are responsible for the vectorial uptake of metformin from blood and its efflux into urine, respectively (Song et al. 2008, Tsuda et al. 2009a; Glucophage® label).

Drug‐drug interactions (DDIs) with metformin are of clinical concern as elevated plasma concentrations of metformin are associated with an increased risk of lactic acidosis (Glucophage^®^ label, Stage et al. 2015). DDIs resulting in less than a 2‐fold increase in the plasma exposure (AUC) of metformin have been observed clinically between metformin and cimetidine (Somogyi et al. 1987; Wang et al. 2008), metformin and trimethoprim (Grün et al. 2013; Müller et al. 2015) and metformin and pyrimethamine (Kusuhara et al. 2011), and are all accompanied by a reduction in its active renal elimination attributed to inhibition of renal organic cation transport. Initially, inhibition of OCT2 was deemed responsible for the observed clinical interactions perpetrated by these drugs (Wang et al. 2008). However, the finding that these drugs were more potent inhibitors of MATE transporters compared to OCT2 shifted opinion to inhibition of MATE1 and/or MATE2‐K as the primary cause of the majority of metformin DDIs (Tsuda et al. 2009b; Ito et al. 2012; Hillgren et al. 2013).

Despite this revised knowledge and using mechanistic static equations putting [I]/*K*_i_ into context with transporter ƒ_e_ (Elsby et al. 2012, 2016), it has not so far been possible to reconcile predicted maximum theoretical metformin AUC increases due to renal transporter inhibition with the clinically observed AUC increases, with the former usually over‐predicting the latter using inhibitor *K*_i_ values reported in the literature. As a consequence of this, and coupled with the observation from AUC profiles of metformin in the presence of interacting drugs that suggest a possible increase in absorption, it is conceivable that an interaction at the level of the intestine may contribute to observed metformin DDIs. Based on studies in polarized Caco‐2 cell monolayers, metformin crosses into enterocytes via transporters, primarily OCT1 located on the apical brush‐border membrane, but once inside the cell has limited basolateral efflux and so is not absorbed transcellularly (Han et al. 2013, 2015). Rather, metformin is hypothesised to undergo absorption via the paracellular route between enterocytes, and as the drug transits along the intestinal tract and luminal concentrations decrease, the transporters such as OCT1 reverse direction and release metformin down its concentration gradient back into the gut lumen for further absorption (Proctor et al. 2008). It is this depot phenomenon that likely gives rise to the slow absorption of metformin resulting in it exhibiting “flip‐flop” pharmacokinetics (Yáñez et al. 2011).

In this study, we sought to investigate the in vitro inhibitory potential of cimetidine, trimethoprim and pyrimethamine against metformin transport mediated by the organic cation transporters OCT1, OCT2, MATE1 and MATE2‐K in order to provide IC_50_ (*K*_i_) values that could be used in mechanistic static predictions of theoretical exposure change due to DDI. Furthermore, we assessed whether these same perpetrator compounds could enhance the absorptive permeability of metformin in Caco‐2 cell monolayers, using substrate and inhibitor concentrations relevant to expected clinical intestinal concentrations, in order to investigate whether an intestinal interaction may contribute towards observed DDIs.

## Materials and Methods

### Materials

Metformin, cimetidine, trimethoprim, pyrimethamine, ammonium chloride, sodium butyrate, non‐essential amino acids, MES, human serum albumin (HSA), lucifer yellow and HEPES were purchased from Sigma‐Aldrich (Poole, Dorset, UK). [^14^C]‐Metformin was purchased from American Radiolabeled Chemicals (St Louis, MO) and Optiphase Supermix liquid scintillation cocktail, 24‐well liquid scintillation counting visiplates and 96‐well isoplates were purchased from PerkinElmer Life and Analytical Sciences (Buckinghamshire, UK). Hanks balanced salt solution (Gibco™ HBSS; containing CaCl_2_ and MgCl_2_), Dulbecco's modified eagle medium (Gibco™ DMEM; high glucose with GlutaMax and pyruvate), fetal bovine serum (Gibco™; heat inactivated) and mammalian protein extraction reagent (M‐PER) were purchased from Fisher Scientific (Loughborough, UK). All other chemicals, solvents, and reagents were purchased from Fisher Scientific.

Biocoat™ Poly‐D‐lysine 24‐well multiwell plates, human organic cation transporter (OCT) 1 (SLC22A1)‐, OCT2 (SLC22A2)‐, multidrug and toxin extrusion protein (MATE) 1 (SLC47A1)‐, and MATE2‐K (SLC47A2)‐expressing TransportoCells™ and vector control cells were supplied by Corning BV Life Sciences (Amsterdam, The Netherlands). Fasted simulated intestinal fluid (FaSSIF) powder was purchased from Biorelevant (London, UK). Millicell‐96 multiwell cell culture insert plates (with polycarbonate membranes; 0.4 *μ*m pore size, 0.12 cm^2^ surface area) and Millicell 96‐well transport companion plates were purchased from Millipore (Watford, Hertfordshire, UK). 96‐Well deep well Abgene polypropylene or shallow round‐bottomed polypropylene plates were supplied by Fisher Scientific. Caco‐2 cells (HTB37, supplied at passage number 17) were obtained from American Type Culture Collection.

### Time linearity and kinetic determination of the transporter‐mediated uptake of metformin by OCT1, OCT2, MATE1, and MATE2‐K

OCT1, OCT2, MATE1, MATE2‐K and control cell lines were seeded in cell culture medium (consisting of DMEM supplemented with 10% (w/v) fetal bovine serum and 1% (v/v) nonessential amino acids) at 3–4 × 10^5^ cells per well in 24‐well poly‐D‐lysine coated plates to achieve a preassay confluence of typically 80–95%. The media was changed 3–4 h postseeding and the cells were cultured at 37°C, 8% CO_2_ for 24 h (media for MATE cells, and corresponding control cells, contained 2 mmol/L sodium butyrate). Prior to the assay, cells were washed twice with prewarmed uptake buffer (HBSS containing 10 mmol/L HEPES, pH 7.4) then left to preincubate in warm uptake buffer for 10 min (MATE cells and corresponding control cells were preincubated in warm uptake buffer solution containing 40 mmol/L ammonium chloride for 20 min). After the preincubation period, uptake buffer was removed and the appropriate incubation solutions were added to the wells. Incubation solutions contained ≤1.2% (v/v) DMSO. For time linearity experiments performed in transporter‐expressing cells and corresponding control cells (triplicate wells per condition), [^14^C]‐metformin (100 *μ*mol/L; 37 kBq/mL) was incubated at 37°C for 1, 2, 3, 5, 10 and 15 min with OCT1, MATE1 or MATE2‐K cells, and 2, 5, 10, 15, 30, and 45 min with OCT2 cells. At the end of the incubation, active transport processes were terminated by removing (via aspiration) the incubation solutions, immediately washing the cells twice with ice cold uptake buffer and then placing plates on ice. Following the wash steps, M‐PER (400 *μ*L) was added to each well and cells were lysed for at least 5 min at 250 rpm on an orbital shaker. An aliquot (300 *μ*L) of cell lysate was added to a white walled, clear bottomed 24‐well visiplate, liquid scintillation cocktail (2 mL) was added, and samples were counted on a Microbeta2 scintillation counter (PerkinElmer) in order to determine the total radioactivity (disintegrations per minute; dpm) taken up in cells. Separately, the protein content of cell lysates (25 *μ*L) was determined using a Bicinchoninic acid (BCA) protein assay kit according to the manufacturer's instructions.

Using the optimum linear incubation time determined above (OCT1 = 10 min; OCT2 = 5 min, MATE1/2‐K = 1.5 min), the uptake of [^14^C]‐metformin into transporter‐expressing cells and vector control cells was determined at 37°C over a range of concentrations (10, 30, 100, 300, 1000, 3000 and 10,000 *μ*mol/L, OCT1 and MATE1/2‐K; 1, 10, 100, 300, 1000, 3000 and 10,000 *μ*mol/L, OCT2) in order to determine an apparent *K*_m_.

### In vitro SLC transporter inhibition assessment

Incubations were performed as described above using the optimum linear incubation times stated for the K_m_ determinations and a metformin probe substrate concentration that was at least 10× lower than the apparent K_m_ determined for each respective transporter.

For OCT1 assessment, uptake of the probe substrate [^14^C]‐metformin (100 *μ*mol/L) was determined (in triplicate wells per condition, over three separate occasions) at 37°C in OCT1‐expressing cells and vector control cells, in the absence and presence of cimetidine (1‐3000 *μ*mol/L), trimethoprim (1‐300 *μ*mol/L) or pyrimethamine (0.3‐100 *μ*mol/L). For OCT2 assessment, uptake of the probe substrate [^14^C]‐metformin (100 *μ*mol/L) was determined (in triplicate wells per condition, over three separate occasions) at 37°C in OCT2‐expressing cells and vector control cells, in the absence and presence of cimetidine (3‐3000 *μ*mol/L), trimethoprim (1‐1000 *μ*mol/L) or pyrimethamine (0.3‐100 *μ*mol/L). For MATE1 assessment, uptake of the probe substrate [^14^C]‐metformin (100 *μ*mol/L) was determined (in triplicate wells per condition, over three separate occasions) at 37°C in MATE1‐expressing cells and vector control cells, in the absence and presence of cimetidine (0.1‐100 *μ*mol/L), trimethoprim (0.1‐100 *μ*mol/L) or pyrimethamine (0.003‐1 *μ*mol/L). For MATE2‐K assessment, uptake of the probe substrate [^14^C]‐metformin (100 *μ*mol/L) was determined (in triplicate wells per condition, over three separate occasions) at 37°C in MATE2‐K‐expressing cells and vector control cells, in the absence and presence of cimetidine (0.1‐100 *μ*mol/L) or trimethoprim (0.03‐30 *μ*mol/L).

### Data analysis

The determined total uptake of probe substrate [^14^C]‐metformin into cells (pmol) was normalised to the protein (mg) content of each well to calculate the uptake activity (pmol/mg). For *K*_m_ determination experiments the calculated uptake activity (pmol/mg) was also normalized for incubation time (min) to give uptake rate (pmol/min/mg). Uptake rate of probe substrate into transporter‐expressing cells was corrected for that determined into vector control cells to calculate the transporter‐mediated (corrected) uptake rate. For *K*_m_ determinations, corrected uptake rate (pmol/min/mg) was plotted against nominal substrate concentration and fitted using SigmaPlot 12.5 (Michaelis–Menten equation). For IC_50_ determinations, corrected uptake activity (pmol/mg) was converted to percentage (vehicle) control activity, which was subsequently plotted against nominal inhibitor concentration. Curves were fitted using SigmaPlot 12.5 (four‐or five parameter logistic equation) to determine the concentration that produces half‐maximal inhibition of probe substrate transport (IC_50_).

### Assessment of metformin apparent permeability across polarised Caco‐2 cell monolayers in the absence and presence of cimetidine, trimethoprim, and pyrimethamine

Caco‐2 cells between passage numbers 50–60 were used for experiments. Cells were seeded in cell culture medium (consisting of DMEM supplemented with 10% (w/v) fetal bovine serum, 2 mmol/L l‐glutamine, 1% (v/v) nonessential amino acids, 50 U/mL penicillin and 50 *μ*g/mL streptomycin) onto Millicell‐96 multiwell insert plates at 1 × 10^5^ cells/cm^2^ and cultured at 37°C in an atmosphere of 5% CO_2_ with a relative humidity of 95 %. Media was changed every two or three days and plates were used for transport studies on day 20 postseeding. Cell monolayers were washed twice with prewarmed (37°C) transport buffer (HBSS containing 25 mmol/L HEPES and 4.45 mmol/L glucose, pH 7.4) for basolateral compartments and prewarmed MES‐transport buffer (HBSS containing 10 mmol/L MES and 4.45 mmol/L glucose, pH 6.5) for apical compartments. Cells were then preincubated with transport buffer (basolateral compartments) or MES‐transport buffer (apical compartments) for 40 min at 37°C prior to the addition of donor and receiver solutions.

Following this preincubation period, donor solutions of transport buffer, or MES‐transport buffer, containing [^14^C]‐metformin with or without test inhibitor were added to basolateral (total volume = 210 *μ*L) or apical compartments (total volume = 90 *μ*L), respectively. Donor compartments also contained the cell monolayer integrity marker lucifer yellow (100 *μ*mol/L). Transport buffer or MES‐transport buffer containing DMSO or test inhibitor was added to the corresponding receiver compartments. Donor and receiver solutions contained a final DMSO concentration of ≤1 % (v/v). Following a 90‐min incubation at 37°C, receiver compartments were sampled (50 *μ*L) into 96‐well isoplates (followed by the addition of 200 *μ*L liquid scintillation cocktail) and the amount of [^14^C]‐metformin was quantified by liquid scintillation counting on a Microbeta2 counter, and used to calculate apparent permeability (P_app_), as described previously (Elsby et al. 2008). Mass balance (percent recovery) of metformin was calculated as described previously (Elsby et al. 2008). The amount of lucifer yellow appearing in the receiver compartments was quantified on a fluorescence plate reader and used to calculate permeation across the cell monolayer. Cell monolayer integrity was deemed acceptable for a well if the determined *P*_app_ for lucifer yellow was ≤1.0 cm/sec (× 10^−6^).

The bidirectional [apical‐to‐basolateral (A–B) and basolateral‐to‐apical (B–A)] apparent permeability of [^14^C]‐metformin across Caco‐2 cell monolayers (buffer pH 6.5/7.4; apical/basolateral) was assessed in triplicate wells per incubation condition at metformin concentrations of 10, 100, 1000 and 10,000 *μ*mol/L. Further assessment in buffer (pH 6.5/7.4) was conducted at a single [^14^C]‐metformin concentration of 10,000 *μ*mol/L in the absence and presence of cimetidine (1000 *μ*mol/L), trimethoprim (500 *μ*mol/L) or pyrimethamine (200 *μ*mol/L). Subsequently, this latter experiment was repeated in buffer containing FaSSIF (pH 6.5) and buffer containing 1 % (w/v) HSA (pH 7.4), present in the apical and basolateral compartments, respectively.

### Mechanistic static predictions of AUC changes for known clinical DDIs with metformin based upon determined in vitro OCT2 and MATE1 inhibitory data

The OCT2 and MATE1 IC_50_ (equating to *K*_i_) values obtained for cimetidine, trimethoprim, and pyrimethamine were incorporated into the adapted Rowland‐Matin mechanistic static equation below, as described previously by Elsby et al. (2012, 2016), to predict the change in metformin AUC based upon inhibition of a fraction excreted (ƒ_e_) value of 0.66 for renal OCT2/MATE1:

$$\mathrm{Fold}\;\Delta \;\mathrm{AUC}=\frac{1}{\frac{f_{e}}{\left(1+\left[I\right]/K_{i}\right)}+\left(1-f_{e}\right)}$$

where *K*_i_ = absolute inhibition constant (equating to IC_50_ if the probe [S] ≪≪ *K*_m_ in the inhibition assay and assuming competitive inhibition, based on the Cheng‐Prusoff equation; Cheng and Prusoff (1973)) and [I] = unbound maximum plasma concentration (*C*_max_).

Additionally, the predicted change in metformin AUC based upon inhibition of OCT2/MATE1 with a lower fraction excreted value of 0.39 was also determined. This lower ƒ_e_ value (0.39) is derived from the renal clearance determined in human mass balance studies following oral administration of metformin, whereas the higher ƒ_e_ value (0.66) is derived from the more usual determination of renal clearance contribution following intravenous administration.

## Results

### Time linearity and kinetic determination of the transporter‐mediated uptake of metformin by OCT1, OCT2, MATE1, and MATE2‐K

**Figure 1:**
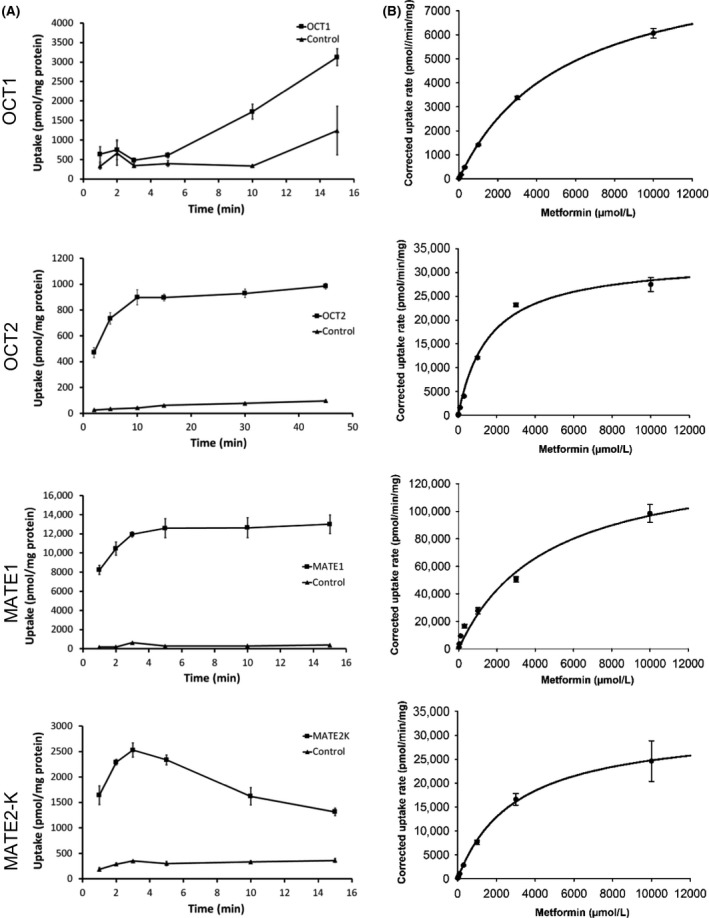
Time linearity (A) and Michaelis–Menten kinetic analysis (B) of metformin transport mediated by OCT1, OCT2, MATE1, and MATE2‐K. Data are expressed as mean (±SD) of triplicate wells per condition.

Uptake of the probe substrate [^14^C]‐metformin (100 *μ*mol/L) into transporter‐expressing cells was linear from 5 to 15 min for OCT1, up to 5 min for OCT2, up to 2 min for MATE1 and up to 2 min for MATE2‐K (Fig. 1), giving acceptable minimum uptake ratios over control cells of 5, 18, 47, and 9, respectively. Concentration‐dependent increases in the transporter‐mediated (corrected) uptake of [^14^C]‐metformin were observed with all transporters, giving apparent *K*_m_ values ranging from 1608 *μ*mol/L for OCT2 to 5422 *μ*mol/L for OCT1 (Table 1 and Fig. 1).

**Table 1:** *K*_m_ and *V*_max_ kinetic parameters for transporter‐mediated uptake of metformin

Transporter	Kinetic parameters (±SE)	
	*K*_m_ (*μ*mol/L)	*V*_max_ (pmol/min/mg protein)
OCT1	5422 ± 369	9370 ± 297
OCT2	1608 ± 175	32918 ± 1165
MATE1	4565 ± 903	141000 ± 12283
MATE2‐K	2986 ± 817	32147 ± 3401

*K*_m,_ Michaelis–Menten constant; SE, standard error from fitting; *V*_max_ maximal velocity of uptake.

### Assessment of cimetidine, trimethoprim, and pyrimethamine as inhibitors of organic cation transporters in vitro

**Figure 2:**
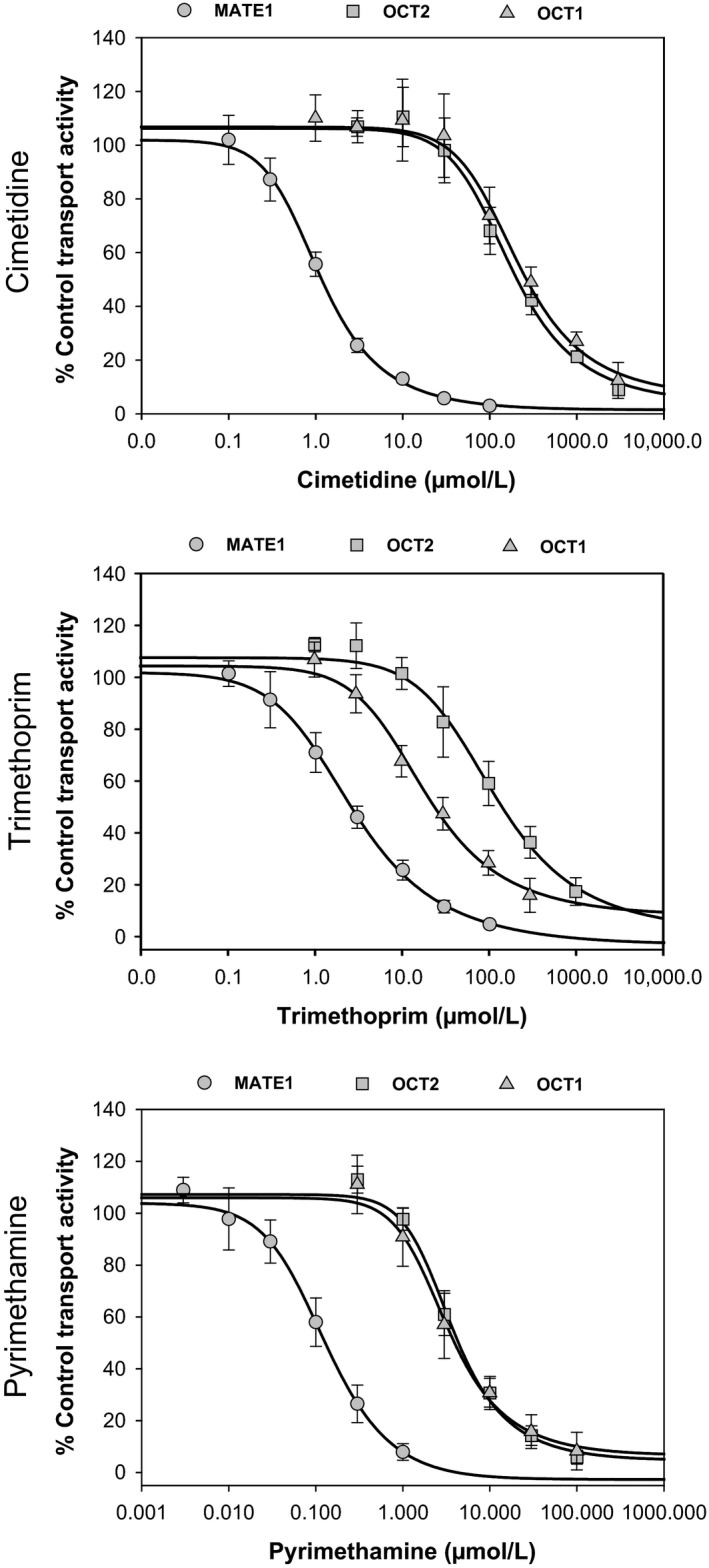
Mean concentration‐dependent inhibition of OCT1‐, OCT2‐, and MATE1‐mediated transport of [^14^C]‐metformin (100 *μ*mol/L) by cimetidine, trimethoprim, and pyrimethamine. Data are expressed as mean (±SD) from a minimum of triplicate wells over three experimental occasions per inhibitor.

Cimetidine, trimethoprim, and pyrimethamine demonstrated concentration‐dependent inhibition of OCT1‐, OCT2‐, MATE1‐ and MATE2‐K‐mediated transport of [^14^C]‐metformin (100 *μ*mol/L) giving the mean IC_50_ curves shown in Figure 2. The determined mean IC_50_ values for each inhibitor from three separate experiments are shown in Table 2. The inhibitory potential (IC_50_) of cimetidine towards MATE1 was approximately 225‐fold and 170‐fold more potent than towards OCT1 and OCT2, respectively. A similar trend was observed for trimethoprim which produced a MATE1 IC_50_ that was approximately 10‐fold and 52‐fold more potent compared to OCT1 and OCT2. Of all the inhibitors tested, pyrimethamine gave the lowest IC_50_ versus MATE1 (0.131 *μ*mol/L), which again was more potent (35‐fold) compared with OCT1 and OCT2 transporters.

**Table 2:** IC_50_ values for inhibition of transporter‐mediated uptake of metformin by cimetidine, trimethoprim, and pyrimethamine

Inhibitor	IC_50_ (≅*K*_i_) values (*μ*mol/L) (Mean ± SD)			
	OCT1	OCT2	MATE1	MATE2‐K
Cimetidine	275 ± 32.2	207 ± 19.6	1.22 ± 0.0870	3.34 ± 1.02
Trimethoprim	27.7 ± 3.80	137 ± 58.0	2.64 ± 0.271	0.353 ± 0.0584
Pyrimethamine	4.46 ± 0.770	4.55 ± 1.12	0.131 ± 0.0401	N.D.

IC_50,,_ half maximal inhibitory concentration; *K*_i,,_ absolute inhibition constant (equates to IC_50_ in these assays as probe substrate concentration utilized is ≪≪*K*_m_); ND, not determined.

Assessment of DDI potential versus intestinal OCT1 inhibition using FDA static equation approaches gave theoretical intestinal luminal concentration [I_2_]/*K*_i_ ratios of 23, 99, and 180 for cimetidine (400 mg dose; [*I*_2_] = 6341 *μ*mol/L), trimethoprim (200 mg dose; [*I*_2_] = 2756 *μ*mol/L) and pyrimethamine (50 mg dose; [*I*_2_] = 804 *μ*mol/L), respectively. Determined *C*_max free_/*K*_i_ ratios for the inhibition potential of cimetidine, trimethoprim, and pyrimethamine toward OCT2 and MATE1 are shown in Tables 3 and 4, respectively, and for all inhibitors were less than 0.1 for OCT2, or greater than 2 (>0.1) for MATE1.

**Table 3:** Predicted versus observed AUC increases of metformin with various coadministered drugs based upon inhibition of renal OCT2

Perpetrator drug	Dose (mg)	*f*_u_	[*C*_max_] (*μ*mol/L)	[*C*_max free_] (*μ*mol/L)	OCT2 *K*_i_ (*μ*mol/L)	[*C*_max free_]/*K*_i_ ratio	Predicted AUC fold‐increase (ƒ_e _= 0.66)	Predicted AUC fold‐increase (ƒ_e _= 0.39)	Observed AUC fold‐increase (metformin dose mg)
Cimetidine	400	0.80^a^	9.6 5.1	7.68 4.08	207	0.037 0.020	1.02 1.01	1.01 1.01	1.46 (250 mg)^b^ 1.54 (500 mg)^c^
Trimethoprim	200	0.56	14^d^ 7.6	7.84 4.26	137	0.057 0.031	1.04 1.02	1.02 1.01	1.37 (500 mg)^e^ 1.30 (850 mg)^f^
Pyrimethamine	50	0.13	2.29	0.298	4.55	0.065	1.04	1.02	1.39 (250 mg)^g^

*f*_u_, fraction unbound (taken from the Bactrim and Daraprim drug labels accessed via Drugs@FDA database; https://www.accessdata.fda.gov/scripts/cder/daf); *C*_max_ Mean steady‐state maximum plasma concentration for total (bound plus unbound) drug measured in the clinical interaction study with metformin; K_i_ absolute inhibition constant (assuming competitive inhibition; equates to IC_50_ in these assay as probe substrate concentration utilised is ≪≪*K*_m_).

a Somogyi and Gugler (1983).

b Somogyi et al. (1987).

c Wang et al. (2008).

d Hruska et al. (2005).

e Grün et al. (2013).

f Müller et al. (2015).

g Kusuhara et al. (2011).

**Table 4:** Predicted versus observed AUC increases of metformin with various coadministered drugs based upon inhibition of renal MATE1

Perpetrator drug	Dose (mg)	*f*_u_	[*C*_max_] (*μ*mol/L)	[*C*_max free_] (*μ*mol/L)	MATE1 *K*_i_ (*μ*mol/L)	[*C*_max free_]/*K*_i_ ratio	Predicted AUC fold‐increase (ƒ_e _= 0.66)	Predicted AUC fold‐increase (ƒ_e _= 0.39)	Observed AUC fold‐increase (metformin dose mg)
Cimetidine	400	0.80^a^	9.6 5.1	7.68 4.08	1.22	6.30 3.34	2.32 2.03	1.51 1.43	1.46 (250 mg)^b^ 1.54 (500 mg)^c^
Trimethoprim	200	0.56	14^d^ 7.6	7.84 4.26	2.64	2.97 1.61	1.98 1.69	1.41 1.32	1.37 (500 mg)^e^ 1.30 (850 mg)^f^
Pyrimethamine	50	0.13	2.29	0.298	0.131	2.27	1.85	1.37	1.39 (250 mg)^g^

*f*_u_, fraction unbound (taken from the Bactrim and Daraprim drug labels accessed via Drugs@FDA database; https://www.accessdata.fda.gov/scripts/cder/daf))

*C*_max_, Mean steady‐state maximum plasma concentration for total (bound plus unbound) drug measured in the clinical interaction study with metformin; *K*_i,_ absolute inhibition constant (assuming competitive inhibition; equates to IC_50_ in these assay as probe substrate concentration utilized is ≪≪*K*_m_)

a Somogyi and Gugler (1983)

b Somogyi et al. (1987)

c Wang et al. (2008)

d Hruska et al. (2005)

e Grün et al. (2013)

f Müller et al. (2015)

g Kusuhara et al. (2011)

### Effect of cimetidine, trimethoprim, and pyrimethamine on the apparent permeability of metformin across polarised Caco‐2 cell monolayers

**Figure 3:**
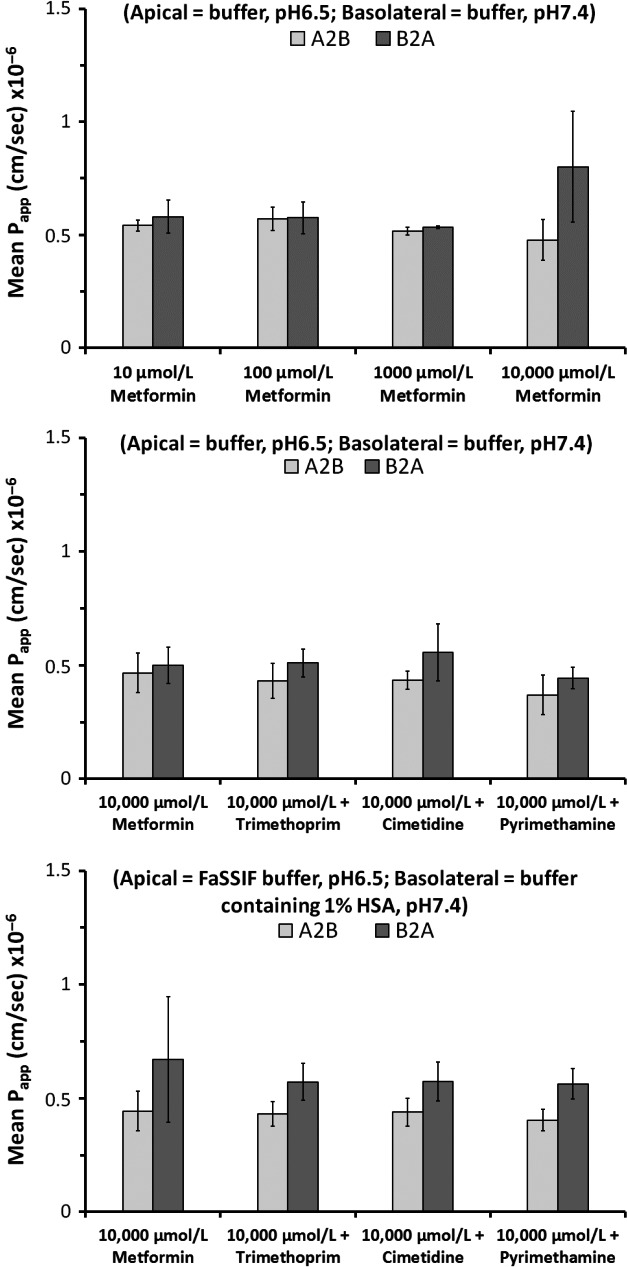
Mean bidirectional apparent permeability of a range of concentrations of metformin (10‐10,000 *μ*mol/L), and of a single concentration of metformin (10,000 *μ*mol/L) in the absence and presence of cimetidine (1000 *μ*mol/L), trimethoprim (500 *μ*mol/L) or pyrimethamine (200 *μ*mol/L), across polarized Caco‐2 cell monolayers at pH6.5/7.4. Incubations with inhibitors were conducted both in buffer alone or buffer containing FaSSIF and 1 % (w/v) human serum albumin. Data are expressed as mean (±SD) of *n* =3–8 wells per condition.

The determined bidirectional P_app_ (cm/sec × 10^−6^) of a range of concentrations of metformin in buffer (pH6.5/7.4) is shown in Figure 3 and was approximately 0.5 cm/sec (× 10^−6^) in both the A‐B and B‐A directions across all metformin concentrations (10–10,000 *μ*mol/L). Furthermore, there was no change to the determined A‐B or B‐A P_app_ values of metformin (10,000 *μ*mol/L) in the presence of cimetidine, trimethoprim or pyrimethamine when incubations were conducted either in buffer alone (pH6.5/7.4), or in FaSSIF containing buffer (pH6.5) apically with 1 % HSA (w/v) containing buffer (pH 7.4) basolaterally (Fig. 3).

### Predicted versus observed AUC changes for known clinical DDIs with metformin based upon determined in vitro OCT2 and MATE1 inhibitory data

Using the drug inhibitory affinities determined above for cimetidine, trimethoprim, and pyrimethamine versus OCT2 and their pharmacokinetic parameters given in Table 3, calculations were performed with mechanistic static equations in order to predict the theoretical fold‐increase in metformin AUC that would occur as a consequence of these drugs' inhibition of renal OCT2. As anticipated, the calculated theoretical fold increases in exposure due to inhibition of OCT2 (with ƒ_e_ = 0.66 or 0.39) gave AUC increases that were below bioequivalence (<1.25; ranging from 1.01–1.04) indicating no DDI potential via this mechanism.

In contrast, the calculated theoretical fold increases in AUC due to inhibition of renal MATE1 by cimetidine, trimethoprim, and pyrimethamine were 2.32/2.03, 1.98/1.69, and 1.85 using an ƒ_e_ value of 0.66, or 1.51/1.43, 1.41/1.32, and 1.37 using an ƒ_e_ value of 0.39, respectively. Predicted AUC increases were within 39‐86 % or 2‐11 % of clinically observed AUC increases when using an ƒ_e_ value of 0.66, or an ƒ_e_ value of 0.39, respectively.

## Discussion

Metformin is widely prescribed to patients due to the prevalence of diabetes as a comorbidity in many diseases (Bloomer et al. 2013). As a consequence of being a common comedication, and because of concern that elevated plasma concentrations are associated with an increased risk of lactic acidosis (Glucophage^®^ label, DeFronzo et al. 2016), there is a requirement to assess the potential for drugs to perpetrate DDIs with metformin during development (Giacomini et al. 2010; Hillgren et al. 2013). Such assessment involves initial in vitro evaluation of the drug as an inhibitor of transporters, followed (if warranted) by a clinical interaction study (US FDA draft DDI guidance 2012). Pivotal to assessment is the prior understanding of the mechanism(s) underlying clinically observed DDIs in order to identify the critical target metformin disposition pathway(s).

Cimetidine, trimethoprim, and pyrimethamine perpetrate clinical DDIs with metformin by reducing its renal clearance through inhibition of renal organic cation transporters thereby increasing the *C*_max_ and AUC of metformin (Somogyi et al. 1987; Wang et al. 2008; Kusuhara et al. 2011; Grün et al. 2013; Müller et al. 2015). In order to confirm the likely mechanism underlying these DDIs, the in vitro inhibitory properties of cimetidine, trimethoprim, and pyrimethamine against OCT2‐, MATE1‐ and MATE2‐K‐mediated metformin transport were assessed in transfected cells to generate IC_50_ values that could be incorporated into mechanistic equations for computing AUC increases due to transporter inhibition. Inhibition experiments were conducted over a linear incubation time using a metformin probe substrate concentration that was at least 10‐fold lower than its determined *K*_m_ for transporter‐mediated uptake (Table 1), such that calculated IC_50_ values equate to *K*_i_ (assuming competitive inhibition; Cheng and Prusoff 1973). This is approach is valid as cimetidine, trimethoprim, and pyrimethamine are reported to be competitive inhibitors of MATE1 (Kusuhara et al. 2011; Ito et al. 2012; Müller et al. 2015). Determined IC_50_ (*K*_i_) values (Table 2) versus OCT2, MATE1 and MATE2‐K were in reasonable concordance with literature values and were much lower (35‐ to 170‐fold; Fig. 1) for MATEs versus OCT2 (Kusuhara et al. 2011; Ito et al. 2012; Müller et al. 2015; Shen et al., 2016). However, only inhibition constants for OCT2 and MATE1 were subsequently incorporated into mechanistic equations for predicting metformin AUC increases as the study of Prasad et al. (2016) determined that these, and not MATE2‐K, are the major cationic transporter proteins expressed in human renal proximal tubules; representing 26% and 18% of renal transporter protein. The reported minimal detectable expression of MATE2‐K protein suggests MATE2‐K may have limited functional activity in human kidney. This notion is further supported clinically by the finding that the MATE2‐K selective inhibitor nizatidine did not increase metformin AUC, nor did it reduce renal clearance in an interaction study, despite its unbound *C*_max_/*K*_i_ ratio being 1.05 indicating the potential for a DDI in vivo through inhibition of MATE2‐K (Morrissey et al. 2016). Collectively, these suggest that MATE2‐K is not involved in renal disposition of metformin in humans and that inhibition of MATE2‐K is not important in manifesting metformin clinical DDIs.

Unbound *C*_max_/K_i_ ratios for inhibition of OCT2 by cimetidine, trimethoprim or pyrimethamine were less than 0.1 resulting in calculated fold‐increases in metformin AUC of ≤1.04 (using ƒ_e _= 0.66), confirming that DDI via this mechanism is unlikely in vivo (Table 3). In contrast, unbound *C*_max_/*K*_i_ ratios for inhibition of MATE1 were 3.34‐6.3, 1.61‐2.97 and 2.27 for cimetidine, trimethoprim and pyrimethamine, respectively, confirming inhibition of this pathway as being clinically relevant to observed DDIs (Table 4) and agreeing with published literature (Tsuda et al. 2009b; Ito et al. 2012). Unbound *C*_max_ is the correct concentration to put into context with *K*_i_ as intracellular concentrations of cimetidine and trimethoprim in renal proximal tubule cells are not anticipated to accumulate above, but rather equilibrate with, plasma levels since they are transported substrates of passive facilitative OCT2 (Bactrim™ label, Severance et al. 2017). However, incorporating these ratios and an ƒ_e _= 0.66 resulted in over‐predicted calculated maximum theoretical fold‐increases in metformin AUC due to MATE1 inhibition compared with clinically observed changes (Table 4).

It is difficult to reconcile this disconnect, however, examinations of metformin AUC profiles from DDI studies with cimetidine, trimethoprim, and pyrimethamine show an increase in the initial rising phase of the curve with parallel terminal phases, suggesting that elevated *C*_max_ and AUC could result from increased metformin absorption. This was investigated using polarized Caco‐2 cell monolayers that, like enterocytes, have been demonstrated to express OCT1 functionally on the apical membrane of cells (Han et al. 2013). OCT1 is the major transporter involved in intestinal uptake of metformin, creating a depot for absorption (Han et al. 2015), and would be expected to be inhibited by cimetidine, trimethoprim, and pyrimethamine in vivo based on calculated [*I*_2_]/*K*_i_ ratios (>10), using IC_50_ values from this study (Table 2) which agreed with literature values (Ito et al. 2012; Müller et al. 2015). The determined absorptive (apical to basolateral) permeability (~0.5 cm/sec × 10^−6^) of 10, 100, 1000 and 10,000 *μ*mol/L metformin in Caco‐2 cells (Fig. 3) was consistent with values reported previously by Song et al. (2016) and Proctor et al. (2008). A concentration of 10,000 *μ*mol/L was chosen to investigate the effect of inhibitors on permeability as this reflects metformin's maximum aqueous solubility; lying between the theoretical intestinal concentration (dose in mol/250 mL) of 250 mg and 500 mg doses of metformin. Additionally, inhibitor concentrations chosen ensured (1) that they were greater than theoretical enterocyte concentrations ([*I*_gut max_]; Agarwal et al. 2013) for clinical doses of cimetidine (528 *μ*mol/L), trimethoprim (230 *μ*mol/L) or pyrimethamine (67 *μ*mol/L) and, (2) that they were >3 times determined OCT1 IC_50_ values to give reasonable or complete inhibition of OCT1. Inhibition of OCT1 would act to reduce the apical uptake of metformin making more available for paracellular absorption (Han et al. 2015), yet the observed absence of an effect of cimetidine, trimethoprim or pyrimethamine on enhancing metformin absorptive permeability across Caco‐2 cells in this study suggests that DDI AUC profile changes are not related to increased metformin absorption.

**Figure 4:**
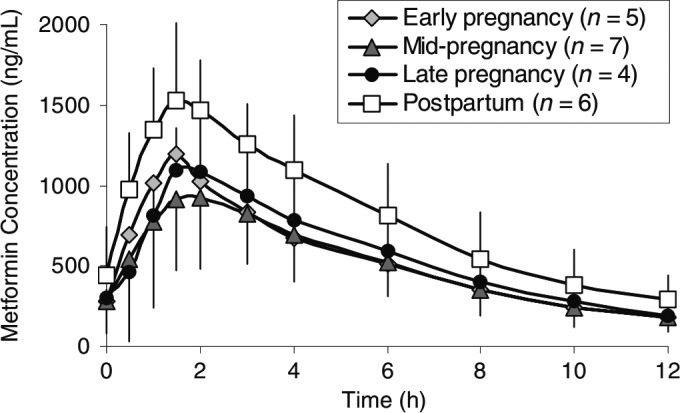
Figure reproduced from Eyal et al. (2010) to illustrate how a documented decrease in metformin (500 mg) renal clearance in pregnancy changes (lowers) the initial rising phase of the AUC profile of a drug (metformin) that exhibits “flip‐flop” pharmacokinetics.

Interestingly, in the clinical scenario where there is increased metformin renal clearance due to pregnancy, the resulting reduction in *C*_max_ and AUC of metformin is accompanied by a lowering of the initial rising phase of the AUC profile without a change in the terminal phase (Eyal et al. 2010; Fig 4). This is consistent with the fact that metformin exhibits “flip‐flop” pharmacokinetics (Tucker et al. 1981), where the rate of absorption of a drug is slower than its rate of elimination, thereby the terminal phase of the AUC profile reflects the absorption rate and the initial rising phase represents the elimination of drug (Yáñez et al. 2011). Consequently, the increase in the initial rising phase of the metformin AUC curve observed in DDIs, giving rise to increased exposure, must result from reduction in renal elimination due to inhibition of MATE1.

This is feasible as inhibition of renal MATE1 would cause intracellular concentrations of metformin to increase, which in turn would act to reduce the blood‐to‐cell concentration gradient of metformin as the driving force for passive facilitative OCT2 transport activity. Consequently, metformin uptake into renal proximal tubule cells would be reduced leading to elevated metformin plasma concentrations as elimination is decreased. This hypothesis was also proposed by Burt et al. (2016) as part of development of a PBPK model towards understanding metformin‐cimetidine DDI. Furthermore, it is supported in vivo by the observation that, following intravenous administration, the 4.2‐fold increase in metformin AUC (giving ƒ_e _= 0.76) in *Mate1* ^*(−/−)*^ null mice compared with wildtype mice (Tsuda et al. 2009a) is identical in effect to the 4.5‐fold increase in metformin AUC (giving ƒ_e _= 0.77) observed in *Oct1/2* ^*(−/−)*^ null mice (Higgins et al. 2012), confirming a complete reduction in Oct1/2 activity towards dual substrates in the absence of functional Mate1.

Having established that increases in metformin AUC observed clinically with cimetidine, trimethoprim and pyrimethamine are due to a reduction in renal clearance via MATE1 inhibition, the over‐prediction of theoretical AUC change needs to be addressed. This is likely attributed to deriving an incorrect ƒ_e_ value (0.66) for MATE1. For conventional oral drugs (whose elimination rate <<<absorption rate) ƒ_e_ values are derived from total plasma clearance obtained in human intravenous balance studies (Elsby et al. 2012). However, with metformin, due to “flip‐flop” pharmacokinetics, it is being renally eliminated from plasma faster than it can be absorbed. Since absorption is still occurring in parallel to active renal elimination, the extent of renal elimination is reduced (compared to intravenous determination) and would therefore derive a lower ƒ_e_ value. Indeed, taking oral metformin human mass balance into account (Pentikäinen et al. 1979; Tucker et al. 1981; Kusuhara et al. 2011), on average 52% of the dose is eliminated in urine, deriving an ƒ_e _= 0.39. Furthermore, using this revised ƒ_e_ value better predicted (within 11% of clinical values) the clinically observed increases in metformin AUC based on inhibition of MATE1 by cimetidine, trimethoprim, and pyrimethamine. For future studies, it would be interesting to see if the model of Burt et al. (2016) still required the 8 to 18‐fold decrease in cimetidine K_i_ to capture the change in metformin kinetics if this revised renal ƒ_e_ value was taken into account.

Such a differential for renal transporter ƒ_e_ has also been observed based on metformin AUC increases of 2.9‐fold and 4.2‐fold in *Oct1/2* ^*(−/−)*^mice, deriving values of 0.66 and 0.76, following oral and intravenous administration, respectively. If such a differential did not exist then the fold increase in metformin AUC in null mice (due to the absence of Oct1/2 protein) compared to wild type mice would be identical regardless of metformin route of administration. Interestingly, oral AUC profiles of metformin in mice showed the same protracted absorption profile (Higgins et al. 2012) as observed in humans. Moreover, the changed oral AUC profile in null mice compared to wildtype mirrored clinical metformin DDI profile changes due to transporter inhibition, that is, an increase in the initial rising phase.

In conclusion, in vitro DDI studies, coupled with mechanistic static approaches for predicting maximal theoretical increases in exposure, indicate solitary inhibition of renal MATE1 as the likely mechanism underlying the metformin AUC increases in observed clinical interactions perpetrated by cimetidine, trimethoprim, and pyrimethamine.

## Authorship Contributions

*Participated in research design*: Elsby; *Conducted experiments*: Chidlaw, Outteridge, Pickering, Radcliffe, and Sullivan; *Performed data analysis*: Chidlaw, Elsby, Outteridge, Pickering, Radcliffe, and Sullivan; *Wrote or contributed to the writing of the manuscript*: Butler, Chidlaw, Elsby, Jones, Outteridge, Pickering, and Radcliffe.

## Disclosure

None declared.
